# Elderly Hepatocellular Carcinoma Patients: Open or Laparoscopic Approach?

**DOI:** 10.3390/cancers12082281

**Published:** 2020-08-14

**Authors:** Jong Man Kim, Sangjin Kim, Jinsoo Rhu, Gyu-Seong Choi, Choon Hyuck David Kwon, Jae-Won Joh

**Affiliations:** 1 Department of Surgery, Samsung Medical Center, Sungkyunkwan University School of Medicine, Seoul 06351, Korea; yjongman21@gmail.com (J.M.K.); khjginigini@hanmail.net (S.K.); jsrrules@gmail.com (J.R.); gyuseong.choi@samsung.com (G.-S.C.); 2 Department of Surgery, Digestive Disease and Surgery Institute, Cleveland Clinic, Cleveland, OH 44195, USA; chd.kwon@gmail.com

**Keywords:** hepatocellular carcinoma, hepatectomy, tumor recurrence, survival, minimally invasive surgery

## Abstract

It remains unclear whether the short-term benefits and long-term outcomes of laparoscopic liver resection (LLR) accrue to elderly patients with medical comorbidities. The aim of the present study was to compare the outcomes between LLR and open liver resection (OLR) in elderly patients (≥65 years) with solitary, treatment-naïve solitary hepatocellular carcinoma (HCC). From January 2013 to August 2017, 256 elderly patients with solitary treatment-naive HCC underwent liver resection. All patients were Child–Pugh class A and older than 65 years. The OLR and LLR groups contained 160 and 96 patients, respectively. The median tumor size in the OLR group was significantly larger than that in the LLR group (3.9 vs. 2.6 cm), but the tumor size did not differ between the two groups after matching. The median operation time, blood loss, transfusion rate, and postoperative complications in the OLR group did not differ from those in the LLR group, but the operation time in the LLR group was longer than that in the OLR group after matching. The median hospitalization in the LLR group was significantly shorter than that in the OLR group. Disease-free survival (DFS) in the LLR group was better than that in the OLR group before and after matching, but the difference was not significant. Patient survival (PS) in the LLR group was similar to that in the OLR group. LLR is feasible and safe for elderly patients with solitary, treatment-naïve HCC. The short- and long-term benefits of LLR are evident in geriatric oncological liver surgery patients.

## 1. Introduction

As life expectancy continues to increase, the number of elderly individuals is constantly growing worldwide [1]. As the general population continues to age, the need for the surgical management of elderly patients increases. Old age is widely considered to be a risk factor for hepatocellular carcinoma (HCC) [2], and a 67% increase in cancer incidence among patients older than 65 years from 2010 to 2030 has been predicted, whereas only an 11% increase is expected among younger people; among those cancers, more than 88% of hepatobiliary malignancies are expected to occur among patients more than 65 years old [3].

Elderly patients frequently present with more comorbidities than young patients, especially cardiovascular and pulmonary diseases, which increases their risk of postoperative complications [4,5]. Many surgeons are reluctant to operate due to the risk of aging, the risk of bleeding during hepatectomy, and the possibility of liver failure after hepatectomy in elderly HCC patients with cirrhosis [6]. Recently, the outcomes of liver resection have improved due to advanced surgical techniques and improved care before and after surgery. As a result, elderly HCC patients with many accompanying diseases have been safely resected [5,7,8]. Today, open hepatectomy is considered a safe and effective treatment for elderly HCC patients [5,6,7,8]. 

As laparoscopic techniques and instruments continue to improve, laparoscopic liver resection (LLR) in HCCs has become easier [9,10,11]. The studies reported to date have confirmed that LLR for HCC and laparoscopic donor hepatectomy are safer than open liver resection (OLR) and have better recovery after surgery [9,10,12,13]. In addition, the results of LLR for liver malignancies including HCC also showed similar results to OLR. [9,10,11]. However, little is known about the effectiveness of LLR compared to OLR in elderly HCC patients [14,15].

The purpose of this study was to evaluate the surgical outcomes of elderly patients (older than 65 years), by comparing the postoperative and long-term outcomes of patients who underwent OLR with those of patients who received LLR. Furthermore, we sought to identify risk factors for the development of HCC recurrence and death after liver surgery. 

## 2. Results

### 2.1. Baseline Characteristics

The basic demographics and preoperative characteristics of the OLR and LLR groups are summarized in Table 1. The median ages of the patients in the OLR and LLR groups were 69 years (range, 65–84 years) and 70 years (range, 65–82 years), respectively. The proportion of males in the OLR group was higher than that in the LLR group (85.0% vs. 72.9%). The median aspartate transaminase (AST) and alkaline phosphatase (ALP) in the OLR group were higher than those in the LLR group. The presence of hypertension, diabetes, heart disease, cerebrovascular accidents, pulmonary disease, etiology, laboratory results, the indocyanine green retention rate at 15 min (ICG-R15), and tumor markers did not differ significantly between the two groups. After propensity score matching, no variables except ALP differed significantly between the two groups.

### 2.2. Perioperative and Pathologic Characteristics

The perioperative characteristics of the two groups are outlined in Table 2. The median tumor size in the OLR group was significantly larger than that in the LLR group (3.9 vs. 2.6 cm; *p* < 0.001). The presence of cirrhosis in the OLR group was lower than that in the LLR group (31.3% vs. 47.9%; *p* = 0.001). The American Society of Anesthesiologists (ASA) classification, previous abdominal operations, major operations, blood loss during operations, red blood cell transfusions during operations, tumor grade 3 or 4, tumor necrosis, complete encapsulation, microvascular invasion, portal vein tumor thrombosis, intrahepatic metastasis, and free resection margin did not differ significantly between the two groups. The median operation time was 222 min (range, 71–599 min) in the OLR group and 239 min (range, 43–590 min) in the LLR group, respectively. However, the median operation time in the OLR group was significantly shorter than that in the LLR group (200 vs. 240 min; *p* = 0.040) after propensity score matching. In addition, the proportion of portal vein tumor thrombosis (PVTT) in the OLR group was lower than that in the LLR group after propensity score matching (0% vs. 7.7%; *p* = 0.014). The median hospital stay in the OLR group was longer than that in the LLR group both before and after propensity score matching. No variables except operation time, PVTT, and hospitalization differed significantly between the two groups after propensity score matching.

The 90-day mortality rate was 0.8% (*n* = 2) in the OLR group and 0.4% (*n* = 1) in the LLR group, which was not a statistically significant difference. The complications are summarized in Table 3. The overall complication rate was 16.9% (*n* = 27) in the OLR group and 10.4% (*n* = 10) in the LLR group (*p* = 0.199). In patients with complications, the incidence of Clavien-Dingo (CD) grade 3 or 4 was 37% (*n* = 10/27) in the OLR group and 40% (*n* = 4/10) in the LLR group (*p* = 0.986). Surgical complications and pulmonary complications in the OLR group did not differ from those in the LLR group.

### 2.3. Tumor Recurrence and Survival

**Figure 1:**
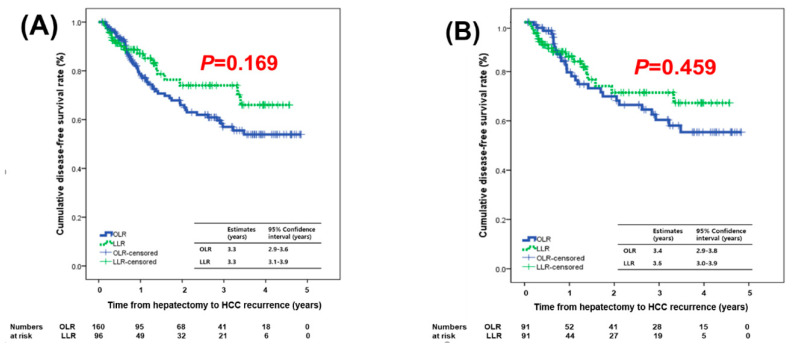
Disease-free survival compared between OLR and LLR groups: (**A**) before matching and (**B**) after matching.

**Figure 2:**
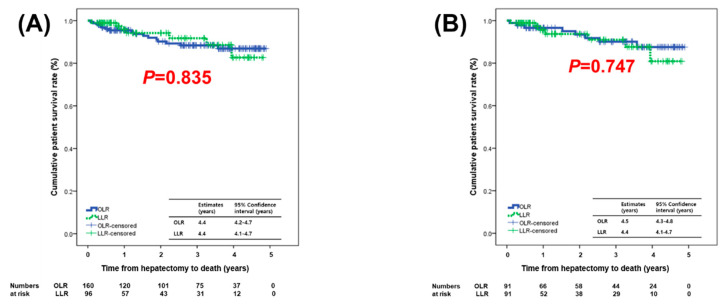
Patient survival compared between OLR and LLR groups: (**A**) before matching and (**B**) after matching.

Before matching, the median follow-up period of the OLR group was 34.9 months (range, 2–58.8 months) and that of the LLR group was 15.8 months (range, 1.8–57.8 months) before matching (*p* = 0.003). Recurrent HCC occurred in 53 patients (33.1%) in the OLR group and 19 (19.8%) in the LLR group during the follow-up period. The initial recurrent site in the OLR group was the liver in 52 patients and peritoneum in one patient. The initial recurrent site in the LLR group was the liver in 18 patients and perihepatic lymph node in one patient. No trocar-site deposits or peritoneal seeding masses were observed in the LLR group. The cumulative disease-free survival (DFS) and patient survival (PS) rates at 1, 2, and 3 years were 78.7%, 65.9%, and 57.0% and 95.5%, 90.2%, and 88.4%, respectively, in the OLR group and 87.0%, 76.4%, and 74.0% and 95.9%, 94.2%, and 91.8%, respectively, in the LLR group. After matching, the cumulative DFS and PS rates at 1, 2, and 3 years were 79.7%, 69.9%, and 58.1% and 96.6%, 93.4%, and 90.1%, respectively, in the OLR group and 86.1%, 71.5%, and 71.5% and 95.6%, 93.7%, and 91.0%, respectively, in the LLR group. The DFS and PS did not differ significantly between the two groups before or after matching (Figure 1 and Figure 2). Interestingly, a trend toward better DFS was observed in the LLR group before and after matching.

The univariate analyses for HCC recurrence and death are summarized in Tables S1 and S2. Multivariate analysis showed that the neutrophil–lymphocyte ratio, ICG-R15, PVTT, and intrahepatic metastasis were closely associated with HCC recurrence before matching. Only red blood cell transfusion during an operation was a predisposing factor for HCC recurrence after matching (Table 4). Age > 75 years, intrahepatic metastasis, long hospitalization, and alpha-fetoprotein (AFP) > 40 were predisposing factors for mortality in the multivariate analysis. After matching, age > 75 years, microvascular invasion, intrahepatic metastasis, red blood cell (RBC) transfusion during an operation, and long hospitalization were closely associated with mortality. LLR was not a risk factor for HCC recurrence or mortality in the univariate and multivariate analyses before or after matching.

## 3. Discussion

As the predicted survival time increases, liver resection in older patients gradually increases [1]. The tolerability of the elderly liver decreases after hepatectomy due to hypofunction and structural changes. Thus, many elderly patients do not receive optimal treatment due to fear of complications after liver surgery [16]. Recently, LLR has been gaining popularity as an alternative to OLR [11,17]. However, it remains uncertain whether elderly patients can obtain the same benefits from LLR as younger patients. This study demonstrated that LLR provides favorable outcomes in elderly patients. 

Blood loss, the transfusion rate, surgical complications, and pulmonary complications did not differ significantly between the OLR and LLR groups before or after propensity score matching (PSM) in this study. LLR is not easy for large tumor volumes over 10 cm. However, we also actively operated on patients with high tumor volumes; thus, there was no difference between the two groups before and after PSM. The operation times in the LLR group were longer than those in the OLR group after matching. However, the hospitalization stay was shorter in the LLR group than in the OLR group. In addition, DFS and PS in the LLR group did not differ from those in the OLR group before or after matching.

In Korea, HBV is the most common cause of HCC, so it is natural for HCC to increase in older patients. However, our study showed that the incidence of non-B non-C (NBNC) as the etiology was higher than that of HBV in elderly patients (46.9% vs. 35.1%). We expected that NBNC HCC might be related to non-alcoholic steatohepatitis (NASH) or alcoholic hepatitis. Previous studies reported that patients over 65 years of age developed more diabetes and cardiovascular disease, such as hypertension, coronary disease, and stroke, than the general population [18,19]. Hypertension was reported in 30% of Koreans over the age of 30, and diabetes was reported in 13.7% [18,19]. In HCC patients over 65 years old, 53.5% had hypertension and 35.2% had diabetes in the present study. Based on these results, we think that people over 65 are elderly patients.

Advances in surgical techniques in liver surgery and improvements in postoperative management have expanded the indications for liver resection, making major liver resection safe for elderly patients [8,20]. It is known that liver function after major liver resection in selective elderly patients is similar to that in younger patients [7,8]. Elderly patients who undergo hepatectomy have increased postoperative complications if the operation time is longer in the LLR than in the OLR [21]. However, we found that LLR had a median operation time similar to OLR before matching, though the median operation time in the LLR group was longer than that in the OLR group after matching (*p* = 0.040). Laparoscopic surgery is a more complex and time-consuming approach than the conventional open approach, but our team has sufficient experience and advanced skills for LLR, so surgical time has ceased to be an obstacle to the spread of LLR in our hospital [17]. 

If bleeding occurs during parenchymal dissection, blood transfusions are needed in the perioperative period. Blood transfusion affects mortality and long-term survival in HCC patients [22]. This study showed that the blood loss and transfusion rate during surgery in the LLR group were similar to those in the OLR group. In addition, the incidence of postoperative complications also did not differ between the two groups. A previous meta-analysis reported that the overall rate of postoperative complications was 21.4% (83/388 cases) for LLR and 33.5% (148/442 cases) for OLR, which was a significant difference. However, we found complication rates of 16.9% (27/160) in the OLR group and 10.4% (10/96) in the LLR group., which was not a significant difference. In addition, the incidence of CD grade 3 or 4 complications in the OLR group did not differ from the rate in the LLR group (6.3% vs. 4.2%). Previous studies have reported that LLR has fewer pulmonary complications than OLR due to less abdominal incision, less muscle division, less postoperative pain, and the preservation of pulmonary function after surgery [11,23]. However, pulmonary complications occurred in eight patients in the OLR group and two patients in the LLR group, so pulmonary complications did not differ between the two groups in our study. In addition, we found that the OLR group had a longer postoperative hospital stay than the LLR group, irrespective of propensity score matching. The limited functional reserves and lower recovery capacity of elderly patients could extend their hospital stays. 

Our elderly patients had a 90-day postoperative mortality rate of less than 1%, which could be associated with the decreased blood loss and decreased surgical wall trauma that occurs with the laparoscopic approach. Previous studies reported that liver resection in the elderly via the open approach had a very high mortality rate ranging from 3.5 to 5.6% [15]. Although the difference in the rate of 90-day postoperative mortality between LLR and OLR was not statistically significant, we did identify a trend favoring LLR, which we attribute to its smaller surgical incision, which would reduce exposure to bacteria and thereby decrease incisional complications.

The outcomes for HCC treated by LLR in elderly patients is the key issue that this study set out to address; therefore, we analyzed the DFS and PS. We found that the OLR and LLR groups had similar DFS and PS rates before and after matching. Interestingly, DFS in the LLR group was better than that in the OLR group even after matching. A previous study showed that worse basic disease conditions and ASA grades predicted poor clinical outcomes [15]; however, those factors had little effect on HCC recurrence or overall survival in our study. We did not identify a significant difference in the survival rate between the OLR and LLR groups. Therefore, pathologic factors, such as PVTT, intrahepatic metastasis, and microvascular invasion, influenced the oncological outcomes more than the surgical approach. 

Although LLR showed similar outcomes to OLR in our results, the liver resection of liver malignancies is still limited in elderly patients with comorbidities [24]. The indication of LLR is almost the same as that of OLR in HCC patients, but LLR should be performed according to tumor location or reserve liver function [9,10,25]. In our study, pathological liver cirrhosis occurred at a rate of 47.9% in the LLR group, but all the hepatectomy patients were Child–Pugh class A.

Several limitations of this study must be considered, including all the limitations inherent in a retrospective analysis. Our patient population exhibits a selection bias because older patients are excluded from patients who receive treatment other than surgery. In addition, there is some selection bias between OLR and LLR despite PS matching because this study is an observational study. In our study, we found big differences in tumor size and follow-up duration. First, the median tumor size in the OLR group was larger than that in the LLR group; therefore, we used propensity score matching to eliminate as much selection bias as possible. Our center considers OLR if the tumor size is 10 cm or more but actively considers LLR if the tumor size is 10 cm or less. Second, the follow-up duration in the OLR group was longer than that in the LLR group. The study period was set from 2013 to 2017, which is the time when our team’s hepatectomy changed from OLR to LLR. Since 2018, most HCC patients over 65 years of age have progressed to LLR, so the baseline characteristics are too different from those for OLR, making it difficult to compare. Currently, our team performs, annually, 600–900 cases of liver resection. Almost 70% of these are LLR. With this experience, tumor location, vascular rapports, and anterior or posterior positions are no longer considered when performing LLR. Because we have performed more than 100 laparoscopic donor liver resections each year, the surgical complexity does not preclude LLR. 

## 4. Materials and Methods

### 4.1. Patients

This study included elderly patients who underwent surgical resection for solitary, treatment-naïve HCC between January 2013 and August 2017. This study was approved by the Samsung Medical Center Institutional Review Board (IRB) (2020-04-068). The requirement for informed consent was exempted by the IRB. The HCC diagnosis was confirmed by pathology reports after hepatectomy. Patients with Child–Pugh class A, solitary, treatment-naïve HCC who were aged ≥ 65 years old before surgery were included. Patients with a history of portal vein embolization or locoregional therapies such as liver resection, radiofrequency ablation (RFA), transarterial chemoembolization, or radiation; multiple tumors; cholangiocarcinoma (CCC); combined HCC and CCC; concurrent intraoperative RFA; R1 resection; serosal involvement; ruptured HCC; synchronous abdominal operations due to other malignant diseases; robotic liver resection; or open conversion cases because of uncontrolled bleeding during a laparoscopic procedure were excluded. A total of 256 suitable patients was identified. Two clinically comparable groups of patients were studied: those undergoing LLR (*n* = 96) and those undergoing OLR (*n* = 160). All the hepatectomies in this study were conducted by one of four surgeons. The selection criteria for using the laparoscopic approach were surgeon dependent. When considering LLR, tumor location, history of portal vein embolization, and trisectionectomy were not considered. The indications for OLR included a tumor size > 10 cm except when a pedunculated type, the reconstruction of a vascular or biliary conduit, proximity to an important vital structure that is deemed difficult to dissect laparoscopically, invasion of adjacent organs that necessitates concomitant resection and reconstruction, future remnant liver < 25%, and Child–Pugh class B. One surgeon did not perform any laparoscopic surgeries, but the other three surgeons used both approaches. 

### 4.2. Clinical Data

Demographic, preoperative laboratory, and pathologic data were prospectively collected from electrical medical records. The collected sociodemographic characteristics were age, sex, body mass index (BMI), and etiology for HCC. Clinicopathologic characteristics included the American Society of Anesthesiologists (ASA) score, comorbidities, and a history of previous abdominal surgery. The surgical factors were blood loss, blood transfusion, the extent of the liver resection, the operative time, the length of the postoperative hospital stay, and postoperative complications for short-term outcomes. Minor liver resection was defined as the removal of one or two segments, and a major liver resection removed three or more segments. Postoperative pathological assessments included tumor size, tumor grade, encapsulation, intrahepatic metastasis, multicentric occurrence, microvascular invasion, and cirrhosis. Postoperative complications were classified according to the Clavien–Dindo (CD) grading system [26] and recorded for up to 30 days or during the same hospitalization in which the surgery was performed. If a patient sustained more than two complications, only the highest one was considered in the data analysis. Death within 90 days of surgery was considered perioperative mortality. None of the patients received postoperative adjuvant therapy before recurrence. The procedures used for surveillance after liver resection have been described previously [27].

### 4.3. Surgical Techniques in Liver Resection

Because this study is retrospective, the surgical methods were selected based on the preferences of individual patients and their surgeons. Our LLR surgical technique was described previously [8,9,12]. After making a pneumoperitoneum with 11–12 mmHg, the intraperitoneal structure was checked using a flexible laparoscopic camera. During the parenchymal dissection, various energy devices were used depending on the surgeon’s preference, and an advanced bipolar device was used for bleeding control. During anatomical resection, the liver was resected by clamping the corresponding Glissonean capsule [28]. Specimens were removed via a small, low–midline incision just below the umbilical port trocar site.

OLR was performed by incision with a J shape or inverted L shape. A Cavitron Ultrasonic Surgical Aspirator (CUSA) (EXcel; Valleylab, Boulder, CO, USA) was used for liver resection, and intermittent inflow control was used to minimize bleeding when necessary. 

### 4.4. Perioperative Management

Each patient received standardized pre- and postoperative management according to the routine manual. The preoperative evaluation of elderly patients did not differ from that used for young patients. Specific tests such as echocardiography, myocardial perfusion scans, or pulmonary function tests were performed only in individual cases, as deemed necessary. All patients wore anti-embolic stockings from before surgery until active ambulation after surgery as a prophylaxis against deep vein thrombosis. All patients received intravenous patient-controlled analgesia using fentanyl after surgery, and additional analgesic drugs were administered to meet the needs of individual patients in managing postoperative pain. Oral feeding was initiated at postoperative day 1, and the diet progressed step by step from water to other liquids to a soft diet, as tolerated by each patient. Patients were discharged once they were free of complications.

### 4.5. Statistical Analyses

Statistical analysis was performed using SPSS ver. 22.0 (SPSS, Inc., IBM Corporation, Armonk, NY, USA) and R 3.2.1 (Vienna, Austria; http://www.R-project.org/). The continuous variables were median and range, and the nominal variables were number and percent. Continuous variables were compared using the Mann–Whitney U test, and nominal scales were compared using Fisher’s exact test. The differences in DFS and PS between the two groups were compared by the Kaplan–Meier survival method. To eliminate selection bias, PS matching between the OLR group and the LLR group was performed using nearest neighbor matching with a caliper with a width of 0.01 and without [29]. For PS matching, the R program (Vienna, Austria; http://www.R-project.org/) was used. The matching variables were tumor size, sex, protein induced by vitamin K absence or antagonist II, and cirrhosis. One hundred and eighty-two patients were selected after PS matching. The DFS and PS in the two groups selected by PS matching were compared using generalized estimating equations in the R program. *p* < 0.05 was defined as statistically significant.

## 5. Conclusions

We compared the outcomes of LLR and OLR in elderly patients with solitary, treatment-naïve HCC. Although elderly patients have a high incidence of comorbidities, LLR can be safely performed to treat HCC because it poses a similar risk of postoperative complications and a faster postoperative recovery than OLR. Nonetheless, further studies are warranted to evaluate the association between postoperative complications and LLR and to elucidate its role in geriatric oncological liver surgery.

## Figures and Tables

**Table 1:** Baseline characteristics.

Variables	Before PS Matching			After PS Matching		
	OLR (*n* = 160)	LLR (*n* = 96)	*p*-Value	OLR (*n* = 91)	LLR (*n* = 91)	*p*-Value
Sex (male)	136 (85.0%)	70 (72.9%)	0.023	70 (76.9%)	68 (74.7%)	0.863
Age > 75	25 (15.6%)	12 (12.5%)	0.583	12 (13.2%)	11 (12.1%)	0.823
Diabetes	52 (32.5%)	38 (39.6%)	0.280	30 (33.0%)	37 (40.7%)	0.357
Hypertension (HTN)	84 (52.5%)	53 (55.2%)	0.699	50 (54.9%)	50 (54.9%)	1.000
Heart disease except HTN			0.921			0.391
None	149 (93.1%)	89 (92.7%)		84 (92.3%)	85 (93.4%)	
Myocardial infarction	1 (0.6%)	1 (1.0%)		0 (0%)	1 (1.1%)	
Angina pectoris	7 (4.4%)	5 (5.2%)		5 (5.5%)	5 (5.5%)	
Arrhythmia	3 (1.9%)	1 (1.0%)		2 (2.2%)	0 (0%)	
Cerebrovascular accident	3 (1.9%)	1 (1.0%)	0.603	0 (0%)	1 (1.1%)	0.316
Pulmonary disease			0.257			0.180
None	147 (91.9%)	91 (94.8%)		81 (89.0%)	86 (94.5%)	
Asthma	6 (3.8%)	0 (0%)		5 (5.5%)	0 (0%)	
COPD	2 (1.3%)	3 (3.1%)		1 (1.1%)	3 (3.3%)	
Lung cancer	2 (1.3%)	0 (0%)		1 (1.1%)	0 (0%)	
Pneumonia	1 (0.6%)	0 (0%)		1 (1.1%)	0 (0%)	
Pneumoconiosis	1 (0.6%)	0 (0%)		1 (1.1%)	0 (0%)	
Pneumothorax	0 (0%)	1 (1.0%)		0 (0%)	1 (1.1%)	
Tuberculosis	1 (0.6%)	1 (1.0%)		1 (1.1%)	1 (1.1%)	
Etiology			0.097			0.764
NBNC	84 (52.5%)	36 (37.5%)		36 (39.6%)	34 (37.4%)	
HBV	48 (30.0%)	45 (46.9%)		35 (38.5%)	42 (46.2%)	
HCV	21 (13.1%)	12 (12.5%)		14 (15.4%)	12 (13.2%)	
HBV, HCV	2 (1.3%)	1 (1.0%)		2 (2.2%)	1 (1.1%)	
NASH	5 (3.1%)	2 (2.1%)		4 (4.4%)	2 (2.2%)	
WBC	5800 (1730–10,300)	5500 (2370–12,800)	0.219	5640 (1730–9790)	5560 (2370–12,800)	0.875
NLR	0.58 (0.14–2.91)	0.53 (0.09–1.86)	0.415	0.60 (0.15–1.87)	0.52 (0.09–1.86)	0.179
Hemoglobin	13.5 (8.4–16.8)	13.7 (9.6–17.0)	0.418	13.5 (9.7–16.4)	13.7 (9.6–17.0)	0.475
Platelet	174,000 (12,000–710,000)	171,000 (14,000–385,000)	0.401	158,000 (12,000–710,000)	174,000 (14,000–385,000)	0.287
Total bilirubin	0.6 (0.2–1.7)	0.6 (0.2–1.8)	0.713	0.6 (0.2–1.7)	0.6 (0.2–1.9)	0.543
AST	29 (13–147)	25 (14–140)	0.010	29 (15–88)	25 (14–140)	0.061
ALT	26 (5–268)	23 (5–297)	0.051	24 (5–81)	23 (5–297)	0.405
ALP	79 (33–259)	72 (35–393)	0.007	78 (33–259)	71 (35–393)	0.026
INR	1.04 (0.89–1.47)	1.04 (0.90–1.41)	0.408	1.04 (0.90–1.27)	1.04 (0.90–1.41)	0.966
Albumin	4.3 (3.1–5.5)	4.3 (3.2–5.2)	0.395	4.3 (3.1–55)	4.3 (3.2–5.2)	0.639
Creatinine	0.90 (0.48–1.77)	0.91 (0.51–4.21)	0.723	0.89 (0.52–1.77)	0.91 (0.51–4.21)	0.430
CRP	0.12 (0.03–8.38)	0.07 (0.03–4.98)	0.524	0.07 (0.03–3.98)	0.07 (0.03–4.98)	0.919
AFP > 40	46 (30.1%)	35 (36.8%)	0.330	21 (23.1%)	33 (36.3%)	0.074
PIVKA-II > 70	76 (49.4%)	37 (40.7%)	0.233	29 (31.9%)	37 (40.7%)	0.280
ICG-R15	11.3 (2.7–24.5)	10.8 (3.4–44.6)	0.752	11.8 (2.7–24.5)	10.6 (3.4–44.6)	0.431

PS, propensity score; OLR, open liver resection; LLR, laparoscopic liver resection; COPD, chronic obstructive pulmonary disease; NBNC, non-B non-C; HBV, hepatitis B virus; HCV, hepatitis C virus; WBC, white blood cell; NLR, neutrophil–lymphocyte ratio; AST, aspartate transaminase; ALT, alanine transaminase; ALP, alkaline phosphatase; INR, international normalized ratio; CRP, C-reactive protein; AFP, alpha-fetoprotein; PIVKA-II, prothrombin in vitamin K absence-II; ICG-R15, indocyanine green clearance at 15 min.

**Table 2:** Perioperative characteristics.

Variables	Before PS Matching			After PS Matching		
	OLR (*n* = 160)	LLR (*n* = 96)	*p*-Value	OLR (*n* = 91)	LLR (*n* = 91)	*p*-Value
ASA classification			0.949			0.648
1	10 (6.3%)	3 (3.1%)		3 (3.3%)	2 (2.2%)	
2	134 (83.8%)	86 (89.6%)		78 (85.7%)	82 (90.1%)	
3	16 (10.0%)	7 (7.3%)		10 (11.0%)	7 (7.7%)	
Previous abdominal operation	42 (26.3%)	18 (18.8%)	0.223	23 (25.3%)	16 (17.6%)	0.278
Extent of resection (major)	79 (49.4%)	46 (48.4%)	0.898	40 (44.0%)	44 (48.9%)	0.552
Operation time (min)	222 (71–599)	239 (43–590)	0.345	204 (71–408)	240 (43–590)	0.040
Blood loss during operation (mL)	300 (30–3000)	250 (30–2500)	0.750	200 (50–1500)	250 (50–2500)	0.172
RBC transfusion during operation	10 (6.3%)	4 (4.2%)	0.579	1 (1.1%)	4 (4.4%)	0.368
Tumor size (cm)	3.9 (0.3–17.0)	2.6 (0.9–14.0)	<0.001	2.9 (0.3–13.2)	2.6 (0.9–14.0)	0.445
Tumor size > 10 cm	12 (7.5%)	4 (4.2%)	0.425	3 (3.3%)	4 (4.4%)	0.700
Tumor grade 3 or 4	21 (13.3%)	5 (5.2%)	0.053	11 (12.4%)	4 (4.4%)	0.063
Tumor necrosis	67 (43.5%)	35 (38.0%)	0.425	33 (37.9%)	34 (39.1%)	0.876
Complete encapsulation	121 (77.6%)	68 (70.8%)	0.189	63 (72.4%)	64 (70.3%)	0.843
Microvascular invasion	110 (68.8%)	58 (60.4%)	0.178	51 (56.0%)	58 (63.7%)	0.364
Portal vein tumor thrombosis	5 (3.1%)	7 (7.3%)	0.139	0 (0%)	7 (7.7%)	0.014
Intrahepatic metastasis	6 (3.8%)	2 (2.1%)	0.714	3 (3.3%)	2 (2.2%)	0.650
Cirrhosis	50 (31.3%)	46 (47.9%)	0.011	40 (44.0%)	43 (47.3%)	0.766
Free resection margin (mm)	10 (2–55)	10 (2–65)	0.428	10 (2–55)	10 (2–65)	0.865
Hospitalization (day)	10 (5–177)	7 (4–41)	<0.001	9 (5–177)	7 (4–41)	<0.001

PS, propensity score; OLR, open liver resection; LLR, laparoscopic liver resection; ASA, American Standards Association; RBC, red blood cell.

**Table 3:** Complications.

Variables	OLR (*n* = 160)	LLR (*n* = 96)	*p*-Value
Complication	27 (16.9%)	10 (10.4%)	0.199
Clavien–Dindo grade			0.986
1	7	3	
2	10	3	
3	8	3	
4	2	1	
Surgical complications	19 (11.9%)	8 (8.3%)	0.372
Wound	3	0	
Bleeding	2	0	
Biloma	3	2	
Ascites	0	3	
Hematuria	1	0	
Nausea/vomiting	1	0	
Atrial fibrillation	1	0	
Hematemesis	1	0	
Renal dysfunction	2	1	
Delirium	4	0	
Hyperbilirubinemia	1	1	
Cerebrovascular accidents	0	1	
Pulmonary complications	8 (5.0%)	2 (2.1%)	0.329
Pneumonia	2	1	
Atelectasis	2	1	
Pleural effusion	2	0	
Acute respiratory distress syndrome	1	0	
Pulmonary artery embolization	1	0	

LR, open liver resection; LLR, laparoscopic liver resection.

**Table 4:** Risk factors for HCC recurrence and mortality by multivariate analysis.

Variables	Before PS Matching			After PS Matching			
	OR	95% CI	*p*-Value		OR	95% CI	*p*-Value
HCC recurrence							
NLR	2.973	1.436–6.154	0.003	RBC transfusion during operation	3.920	1.030–14.923	0.045
ICG-R15	1.056	1.013–1.101	0.009				
Portal vein tumor thrombosis	3.353	1.271–8.847	0.015				
Intrahepatic metastasis	4.830	1.750–13.330	0.002				
Mortality							
Age > 75	3.426	1.250–9.391	0.017	Age > 75	11.333	2.471–51.982	0.002
Intrahepatic metastasis	16.463	5.948–45.566	<0.001	Microvascular invasion	7.502	1.193–47.167	0.032
Hospitalization	1.030	1.019–1.042	<0.001	Intrahepatic metastasis	29.092	6.627–127.716	<0.001
AFP > 40	3.912	1.472–10.395	0.006	RBC transfusion during operation	13.341	2.466–73.266	0.003
				Hospitalization	1.044	1.026–1.062	<0.001

PS, propensity score; OR, odds ratio; 95% CI, 95% confidence interval; HCC, hepatocellular carcinoma; NLR, neutrophil–lymphocyte ratio; ICG-R15, indocyanine green clearance at 15 min; AFP, alpha-fetoprotein; RBC, red blood cell.

## Data Availability

The datasets generated and analyzed during the current study are not publicly available because the hospital was not allowed to take the datasets out, but they are available from the corresponding author upon reasonable request.

## Supplementary Materials

The following are available online at https://www.mdpi.com/2072-6694/12/8/2281/s1. Table S1: Risk factors for HCC recurrence in univariate analysis; Table S2: Risk factors for mortality in univariate analysis.

## Abbreviations

LLR	laparoscopic liver resection
OLR	open liver resection
HCC	hepatocellular carcinoma
PVTT	portal vein tumor thrombosis
CD	Clavien–Dindo
CUSA	Cavitron Ultrasonic Surgical Aspirator
DFS	disease-free survival
PS	patient survival
NBNC	non-B non-C
NASH	non-alcoholic steatohepatitis
