# Supplementary material for: Elderly Hepatocellular Carcinoma Patients: Open or Laparoscopic Approach?
Source: Cancers (Basel). 2020 Aug 14;12(8):2281. doi: 10.3390/cancers12082281 (PMC7466133; doi:10.3390/cancers12082281)
Supplement: Supplementary file 1 [file cancers-12-02281-s001.pdf]

Supplementary Materials:

Supplement Table 1. Risk factors for HCC recurrence in univariate analysis.

	Before PS matching			After PS matching		
	OR	95% CI	P-value	OR	95% CI	P-value
Sex (female)	0.959	0.535-1.720	0.889	1.027	0.522-2.023	0.938
Age >75 years	1.025	0.525-2.001	0.943	1.418	0.634-3.174	0.395
Etiology (viral)	0.929	0.585-1.477	0.757	1.010	0.563-1.810	0.974
Extent of resection (major)	0.840	0.528-1.336	0.461	0.676	0.371-1.230	0.200
Complete encapsulation	0.768	0.277-2.128	0.612	0.573	0.201-1.637	0.299
Microvascular invasion	1.439	0.883-2.344	0.144	1.187	0.662-2.129	0.564
Portal vein tumor thrombosis	3.586	1.546-8.318	0.003	3.436	1.056-11.185	0.040
Intrahepatic metastasis	4.644	1.861-11.585	0.001	2.363	0.531-9.773	0.235
Cirrhosis	1.417	0.888-2.260	0.144	1.435	0.803-2.563	0.222
LLR	0.693	0.410-1.171	0.171	0.800	0.442-1.448	0.460
Complication	1.237	0.677-2.260	0.489	1.553	0.694-3.476	0.284
Tumor grade 3 or 4	1.061	0.509-2.214	0.874	1.355	0.534-3.436	0.522
Tumor necrosis	1.596	1.002-2.525	0.049	1.357	0.751-2.455	0.312
WBC	0.896	0.775-1.035	0.136	0.835	0.690-1.010	0.063
NLR	2.695	1.540-4.714	0.001	1.981	0.865-4.534	0.106
Hemoglobin	1.072	0.927-1.240	0.349	0.977	0.812-1.175	0.806
Platelet	0.995	0.991-0.999	0.008	0.994	0.989-0.999	0.012
Total bilirubin	0.985	0.908-1.067	0.706	0.976	0.801-1.188	0.806
AST	1.009	1.001-1.017	0.023	1.010	1.000-1.021	0.060
ALT	1.006	1.001-1.011	0.027	1.005	0.998-1.012	0.180
ALP	1.003	0.997-1.010	0.313	1.004	0.996-1.012	0.305
Albumin	0.601	0.329-1.098	0.098	0.241	0.112-0.518	<0.001
Creatinine	0.826	0.394-1.731	0.612	0.517	0.155-1.732	0.285
CRP	0.798	0.560-1.137	0.211	0.835	0.521-1.339	0.454
AFP >40	1.031	0.620-1.714	0.907	1.116	0.595-2.092	0.732
PIVKA-II >70	1.527	0.948-2.459	0.082	1.191	0.642-2.210	0.579
Tumor size	1.036	0.966-1.111	0.323	0.961	0.830-1.112	0.592
ICG-R15	1.057	1.022-1.094	0.001	1.073	1.036-1.111	<0.001
Free resection margin (mm)	0.998	0.979-1.016	0.809	0.996	0.973-1.018	0.699

Operation time	1.000	0.998-1.002	0.958	1.000	0.997-1.003	0.954
Blood loss during operation	1.000	0.999-1.000	0.487	1.000	0.999-1.001	0.705
RBC transfusion during operation	0.902	0.329-2.475	0.842	5.555	1.710-18.051	0.004
Hospitalization	1.005	0.986-1.025	0.593	1.010	0.991-1.029	0.327

* PS, propensity score; OR, odds ratio; 95% CI, 95% confidence interval; LLR, laparoscopic liver resection; WBC, white blood cell; NLR, neutrophil-lymphocyte ratio; AST, aspartate transaminase; ALT, alanine transaminase; ALP, alkaline phosphatase; CRP, C-reactive protein; AFP, alpha-fetoprotein; PIVKA-II, prothrombin in vitamin K absence-II; ICG-R15, indocyanine green clearance at 15 minutes

Supplement Table 2. Risk factors for mortality in univariate analysis.

	Before PS matching			After PS matching		
	OR	95% CI	P-value	OR	95% CI	P-value
Sex (female)	1.118	0.415-3.011	0.826	1.579	0.539-4.623	0.405
Age >75	2.848	1.170-6.937	0.021	5.294	1.992-14.894	0.002
Diabetes	0.479	0.178-1.291	0.146	0.575	0.183-1.807	0.344
Hypertension	0.729	0.322-1.654	0.450	1.513	0.516-4.435	0.451
Cerebrovascular accidents	0.048	0.000-11449.707	0.631	0.049	0.000-1454508711	0.806
Pulmonary disease	2.763	0.814-9.371	0.103	2.198	0.492-9.816	0.302
Etiology (viral)	0.984	0.434-2.232	0.968	0.686	0.248-1.897	0.468
Extent of resection (major)	0.936	0.413-2.122	0.875	1.087	0.394-2.997	0.873
Complete encapsulation	1.135	0.150-8.570	0.902	0.958	0.12107.588	0.968
Microvascular invasion	4.703	1.394-15.872	0.013	4.044	1.134-14.417	0.031
Portal vein tumor thrombosis	4.739	1.397-16.071	0.013	2.595	0.339-19.845	0.358
Intrahepatic metastasis	14.399	5.651-36.690	<0.001	17.877	5.643-56.636	<0.001
Cirrhosis	1.355	0.594-3.092	0.470	1.414	0.512-3.899	0.504
LLR	0.910	0.373-2.218	0.835	1.183	0.426-3.281	0.747
Complication	3.378	1.458-7.827	0.005	6.132	2.218-16.952	<0.001
Tumor grade 3 or 4	2.663	1.048-6.768	0.040	3.153	0.999-9.953	0.050
Tumor necrosis	2.278	0.986-5.265	0.054	2.551	0.908-7.170	0.076
WBC	0.841	0.651-1.087	0.187	0.833	0.603-1.151	0.268
NLR	2.655	1.051-6.708	0.039	1.062	0.216-5.218	0.941
Hemoglobin	0.879	0.691-1.119	0.294	0.751	0.548-1.028	0.074
Platelet	0.999	0.994-1.005	0.786	1.002	0.997-1.007	0.524
Total bilirubin	0.989	0.876-1.115	0.854	0.969	0.422-2.224	0.940
AST	1.007	0.994-1.021	0.296	1.008	0.991-1.026	0.365
ALT	1.001	0.990-1.013	0.811	1.001	0.988-1.015	0.838

ALP	1.009	1.001-1.017	0.035	1.010	1.002-1.018	0.018
Albumin	0.733	0.256-2.104	0.564	0.452	0.126-1.627	0.225
Creatinine	0.641	0.124-3.320	0.596	0.854	0.180-4.062	0.843
CRP	0.768	0.388-1.523	0.450	0.922	0.457-1.859	0.922
AFP >40	4.736	1.930-11.625	0.001	5.026	1.716-14.724	0.003
PIVKA-II >70	2.414	1.012-5.762	0.047	2.740	0.990-7.584	0.052
Tumor size	1.133	1.028-1.249	0.012	1.244	1.072-1.444	0.004
ICG-R15	1.044	0.991-1.101	0.104	1.040	0.978-1.105	0.214
Free resection margin	0.972	0.930-1.016	0.211	0.939	0.877-1.005	0.071
Operation time	1.005	1.001-1.009	0.013	1.006	1.000-1.011	0.039
Blood loss during operation	1.000	1.000-1.001	0.167	1.001	1.000-1.002	0.018
RBC transfusion during op.	3.374	1.144-9.950	0.028	12.232	3.352-44.636	<0.001
Hospitalization	1.028	1.017-1.038	<0.001	1.030	1.018-1.043	<0.001

* PS, propensity score; OR, odds ratio; 95% CI, 95% confidence interval; LLR, laparoscopic liver resection; WBC, white blood cell; NLR, neutrophil-lymphocyte ratio; AST, aspartate transaminase; ALT, alanine transaminase; ALP, alkaline phosphastase; CRP, C-reactive protein; AFP, alpha-fetoprotein; PIVKA-II, prothrombin in vitamin K absence-II; ICG-R15, indocyanine green clearance at 15 minutes
